# Economics of Philanthropy

**Published:** 1898-02-19

**Authors:** 


### ECONOMICS OF PHILANTHROPY.

### II.—THE DANGERS OF "FREE, GRATIS, AND FOR NOTHING."

Our contention, then, is that any charity which tends to make the recipient rely upon it is, to the extent to which he thus relies, pauperising. It may cost people a great effort to ask for alms the first time, but almost none to ask for them again. Mr. McCullagh Torrens, in his book, "The Lancashire Lesson," which deals with the cotton famine at the time of the American war, says, "We cannot help marking the readiness with which, on the first cession of adequate wages, large numbers of people now resort to rates and subscription funds, many of whom three years ago would have shrunk instinctively from such public avowal of indigence." This is the problem of philanthropy: to help the poor in their need without teaching them to rely on such help as a matter of course. It is because poor-law relief seems to contain a pledge which the poor man can at any time call upon the law to fulfil, and which, therefore, tends to keep him from making all the effort he could to support himself, that many economists have condemned it altogether. Mr. Malthus, that much-maligned but most far-seeing man, declares that if there were no such relief, "though there might have been a few more instances of very severe distress, the aggregate mass of happiness among the common people would have been much greater than at present." And so sympathetic a student of social questions as Professor Sidgwick thinks that if State relief were abolished, and the labouring class had, therefore, no source to which they could look for subsistence as a right, they would show a more provident and independent spirit. We venture to differ from both these eminent economists. It seems to us that if there were no poor-law relief it would cause a change in the moral attitude, not of possible recipients, but of donors. It is quite impossible for the average man to inquire personally into every demand made upon his charity; he has his own business to attend to, his own living to make, if he in turn is not to require charity himself. He, therefore, delegates that task to certain officials whom he, with his fellow-citizens, pays for that purpose, and to whom he entrusts the sum necessary to relieve all clamant destitution. He can, therefore, refuse the beggar who pesters him for alms, and feel no shame, be harassed by no doubts as to whether he is, indeed, letting a fellow-creature starve. But if he did not know that there was a fund to which the poor man could resort, he would be much more likely to give heed to these pitiful tales of hunger, to accept as genuine the narrative of the sick wife and the starving children, the impossibility of getting work, and all the other romances which are the beggar's stock in trade. He might know, indeed, that some of them were false, but how would he distinguish these from the true ones? He has not time to spare for investigation, and he has money to spare for relief, and he gives that which costs him least.

We might thus expect to see an enormous increase in the number of beggars, while it is very improbable that we would find any increase in the providence of the poor. All that we know of the improvident, in any class of society, forbids us to think that the fear of imminent, and still less of remote starvation, would teach them to economise. The story of the destitute actress, whose friends subscribed for her enough to keep her for at least a year, and who spent it all in a great entertainment, is hardly an exaggeration of the thoughtlessness of many, both poor and rich. We have seen, on the eve of a strike, the last wages that would be received for an indefinite time spent in rioting and drunkenness. Starvation might be at hand, but hardly one of those whom it threatened would put by a day's wage to provide against it. And, on the other hand, it is not the poor—that is, those who have always had the smallest incomes—who make up the paupers. Nothing is more wonderful than the way in which some of the poor manage to live, to pay their way, and owe no man anything. It may involve the severest self-discipline; but it is such an up-bringing as has sent out many of the strong, self-reliant men who are the pride of our race. Not many great men have sprung of a pauper stock; but many have been the children of the very poor. The difference between the poor man and the pauper is rather a difference of *morale* than of circumstance, and therefore the danger which philanthropists must avoid is doing anything to weaken the *morale* of the independent poor. Let them accentuate and not diminish the difference between the two. How much can be done by making the path of dependence too easy is shown by the success of the famous "Speenhamland Act of Parliament."

The general adoption of the principles involved in it caused this high-sounding name to be given to the well-meant action of some Berkshire justices who resolved, in the year 1795, at the village of Speenhamland, to give grants to those poor men whose wages were too small to keep their families in comfort. These grants were fixed according to the price of wheat and the size of the family. No one was to be sent to the workhouse, work was to be provided for the able-bodied, houses for such as needed them; in short, everything that a man could want was to be given, "free, gratis, and for nothing." No wonder that such a law was well obeyed! A couple married, and went straight from the church to the overseer of poor, to demand the house he was bound to furnish them. In due course they informed him when each successive little pauper might be expected, so that there might be no delay in securing the eighteenpence a week which the baby earned by, like the French king, "giving himself the trouble to be born." Such workmen as preserved their independence were laughed at by their fellows for working for that which was to be had for nothing. Work was given by the parish, indeed, but as the family income did not depend on the work done, it was not executed with any great industry. Yet the farmers liked the plan well enough, chiefly because it enabled them to draw on other people's pockets for the wages of their men. They brought wages lower and lower; and then, even though the rates went up, the landowner, the parson, and the shopkeepers paid a share of the labour by which the farmer alone directly profited.

The reason of this outburst of "Tory Socialism," as M. Emile Boutmy calls it, was not pure philanthropy. The great war with France was just beginning; prices were rising, recruits were wanted; and even young paupers were not to be despised as food for powder, or to fill the places of the older and nobler stock who were killed. We have learnt that, even so, the method was not a wise one; but it is strange that we find the same argument used in France to-day. M. Gide, Professor of Economics at Montpellier, admits that charitable relief is too freely given, and that it will, as Malthus long ago pointed out, tend to increase of population among the non-producers of society, at the expense of the producers. But in the present state of the population of France he justifies it. "It is true," he says, "that the birth-rate is higher in the classes who receive relief than in those who support themselves; but if the children of the former can be made useful citizens the apparent harm would really be a benefit, especially in France, where the wealthy classes either cannot or will not increase their numbers."[1]

Reverting to the Lancashire Cotton Famine, it may be well to point out that so soon as relief works were organised on a proper basis the people, though belonging to the artisan class, settled down to regular work and did the work efficiently and well. Sir Robert Rawlinson, K.C.B., testifies that by recognising the difficulties raised by the problem of providing spade labour for the artisan class a system was evolved which proved in practice successful and satisfactory. This system recognised that an artisan could not be put to spade labour on full time or even half-time at the outset. It was necessary to gradually teach him the work, and in teaching to allow time for the hardening of his hands and the development of his muscles. It was further necessary to see that each man had suitable food, was properly clothed, and that the hours of work were so graduated as to enable an artisan to, step by step, become a skilled labourer. Similar experiments in Paddington during one of the severe winters, and elsewhere under somewhat identical conditions, have proved that the problem of the unemployed in large towns can be adequately met if the lessons and the system which the Lancashire cotton famine evolved are mastered and acted upon. Experiences like these are speedily forgotten, and it is to be regretted that Sir Robert Rawlinson's organisation should have passed into the limbo of forgotten things in view of the constant increase in the population in these islands, and the necessity which must sometimes arise during the winter months of facing the problem of providing work for the unemployed in one or more of our large towns. From an economic point of view, having regard to the quality of the work done and to its cost, Sir Robert Rawlinson's system as pursued during the cotton famine has much to recommend it.

[1] "Principles of Political Economy," Jacobsen's translation, p. 547.

